# Effect of Ground Granulated Blast Furnace Slag Replacement Ratio on Structural Performance of Precast Concrete Beams

**DOI:** 10.3390/ma14237159

**Published:** 2021-11-24

**Authors:** Yong-Jun Lee, Hyeong-Gook Kim, Kil-Hee Kim

**Affiliations:** 1 Department of Architectural Engineering, Kongju National University, 1223-24, Cheonandaero, Seobuk, Cheonan 31080, Korea; lyj8315@kongju.ac.kr (Y.-J.L.); anthk1333@kongju.ac.kr (H.-G.K.); 2 Department of Architectural Engineering & Urban Systems Engineering, Kongju National University, 1223-24, Cheonandaero, Seobuk, Cheonan 31080, Korea

**Keywords:** bond strength, ductility, flexure strength, ground granulated blast furnace slag, precast concrete, shear strength

## Abstract

This study was conducted to investigate the effect of ground granulated blast furnace slag on the structural performance of precast concrete beams, evaluating the flexural, shear and bonding performance by using the replacement ratio of the ground granulated blast furnace slag as a variable. The design strength of the concrete was set at 45 MPa in consideration of the characteristics of precast concrete products, and the replacement ratio of the ground granulated blast furnace slag to replace cement was 30 to 70%. The experimental results showed that all specimens had similar behavioral characteristics regardless of the replacement ratio of the ground granulated blast furnace slag. Comparison of the prediction results obtained by ACI 318-19 and EC 2 showed that the mean flexural strength and shear strength were higher than 1.19 and 1.43, respectively, and the mean bond strength was 1.57, satisfying the required performance. Therefore, the experimental results showed that in using the ground granulated blast furnace slag as an admixture for precast concrete, the cement replacement ratio may be increased up to 70% without causing any problems in securing the structural performance. Summarizing the results of the present study, a ground granulated blast furnace slag replacement ratio of 50% or lower may be reasonably applied.

## 1. Introduction

Since the development of Portland cement in the early 19th century, concrete has been applied to architectural and civil engineering structures as a key structural material because of its simple manufacturing method and excellent durability. And with the advances of the construction industry, many developments and studies have been made to secure the required quality and performance. Various approaches have been proposed to improve concrete quality and performance. In particular, the recycling of industrial byproducts to secure a cement replacement can provide both environmental and economic advantages.

Steel slag, a byproduct from the steel industry, is largely divided according to the production process into blast furnace slag and steel furnace slag. Blast furnace slag, generated from the process of producing pig iron from a blast furnace using iron ore, coke and limestone as raw materials, is further divided into water-cooled slag and air-cooled slag according to the cooling method. The production of blast furnace slag is dependent on the iron-making process, and about 300 kg of blast furnace slag is generally generated when 1 ton of molten steel is produced. Water-cooled slag accounts for about 70–80% of generated blast furnace slag; the rest is air-cooled slag. Blast furnace slag has a chemical composition similar to that of conventional construction materials such as cement and natural aggregate, and its stability allows for recycling into various forms, including cement replacement, admixture, aggregate and bricks [1,2,3,4,5]. As the properties are dependent on the cooling method, water-cooled slag is applied to uses of relatively high added value, such as raw material for cement and admixtures for concrete; however, due to its non-reactivity, air-cooled slag is used as aggregate for roads or aggregate for filling.

According to the results of previous studies, Majhi et al., (2020) [2], Wang and Zhi (2003) [6], Macphee and Cao (1993) [7], Ramezanianpour (2014) [8], Rashad (2018) [9], Jiang et al., (2018) [10] and Haha et al., (2012) [11] showed that concrete manufactured using ground granulated blast furnace slag has advantages of improved rheological properties due to reduced fluidity and hydration heat, increasing the durability in the pozzolanic reaction and enhancing the long-term strength by self-hydration. In addition, Song and Saraswathy (2006) [12], Caijun and Yinyu (1989) [13], Gruskovnjak et al., (2008) [14], Taylor (1997) [15], Shi et al., (2020) [16] and Duży et al., (2021) [17] showed that ground granulated blast furnace slag is known to have excellent workability and high resistance to chemical penetration by acids, seawater and sewage. And Yue et al., (2021) [18] suggest a method for predicting the durability of concrete using ground granulated blast furnace slag. In particular, Özbay et al., (2016) [19] and Kim et al., (2014) [20] reported that the utilization of ground granulated blast furnace slag as a cement replacement has a very significant impact on the environment, because the carbon dioxide emission involved in cement production can be reduced and the cost of concrete production can be saved. However, as reported by Yazıcı et al., (2010) [21], Escalante-Garcia and Sharp (1998) [22] and Lee et al., (2013) [23], since ground granulated blast furnace slag has a latent hydraulic property that causes hydration with cement hydrate as a catalyst, limitation of the replacement ratio and appropriate curing measures are required for successful expression of the initial strength.

As the demand for iron is increasing, the generation of steel slag is increasing every year. Although most steel slag is recycled at present, it is necessary to find high value-added applications to utilize it more positively according to advances of the construction industry. Gradually breaking free from the traditional labor-intensive methods of the construction industry, new construction methods and structural systems are drawing attention to make site management easier and minimize on-site work [24]. Precast concrete is part of these new construction methods; it allows for easy quality control because the members are manufactured in factories.

High-strength concrete is used to increase the durability of precast concrete products, and the application of ground granulated blast furnace slag may effectively improve the workability and chemical resistance of high-strength concrete. In addition, steam curing for expression of the initial strength may resolve the limitation of the replacement ratio of ground granulated blast furnace slag and the problems related to curing, and thus it may allow for extensive recycling. However, research on the structural performance of precast concrete members using ground granulated blast furnace slag is still loose ends.

Therefore, the present study was conducted to investigate the effect of ground granulated blast furnace slag on the structural performance of precast concrete beams in terms of flexural, shear and bonding strength by using the replacement ratio of the ground granulated blast furnace slag as a variable. The experimental results were compared with predictions by ACI 318-19 and EC 2 as well as with results provided in previous reports. Based on this comparison, an appropriate replacement ratio of ground granulated blast furnace slag was investigated to secure structural stability of precast concrete beam members manufactured using ground granulated blast furnace slag. And it is expected that the results of this study can be used as basic data to review the appropriate replacement ratio of ground granulated blast furnace slag in the manufacture of precast concrete members.

## 2. Experimental Program

### 2.1. Materials

Considering the properties of existing precast concrete products, the concrete was designed with a design strength of 45 MPa by varying the replacement ratio of ground granulated blast furnace slag, as shown in Table 1. In the concrete specimen names, the number represents the replacement ratio of ground granulated blast furnace slag. Specimen C4010 is a concrete specimen prepared by substituting ground granulated blast furnace slag and fly ash at ratios of 40% and 10%, respectively. The fineness of the ground granulated blast furnace slag included in the concrete mix was higher than that of cement by about 20% or more. Therefore, as the replacement ratio was increased, the amount of the applied air-entraining agent (AE) was increased to secure the workability of the concrete. Both the coarse and fine aggregates used in the concrete mixing were natural aggregates; their maximum dimensions were 25 mm and 5 mm, respectively.

**Figure 1:**
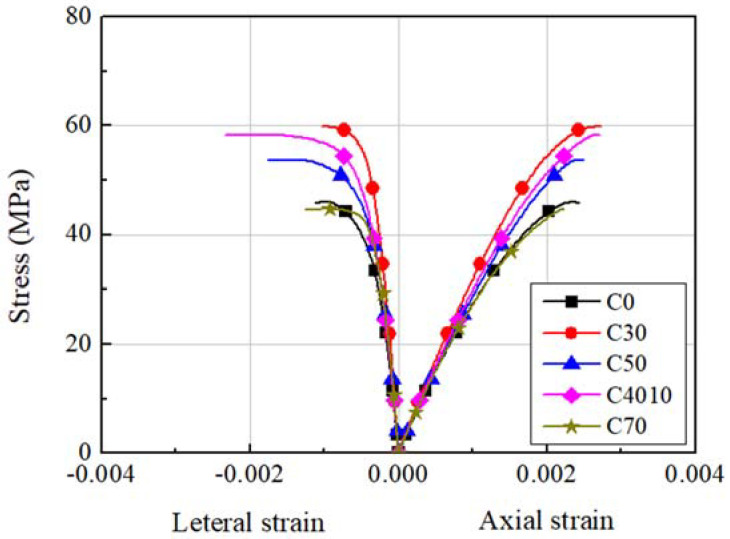
Stress–strain relationships of concrete.

To investigate the compressive strength of the concrete, circular concrete cylinders of Φ100 × 200 mm were prepared when the specimens were placed, and the cylinders were cured under the same conditions as the specimens. The compressive strength of the concrete was tested on the days of experiment for each specimen series; the test results are shown in Figure 1. Regardless of the replacement ratio of the ground granulated blast furnace slag, the compressive strength of the concrete was from 45.2 to 58.0 MPa, higher than the design strength, and the elastic modulus was from 29.3 to 32.6 GPa.

**Figure 2:**
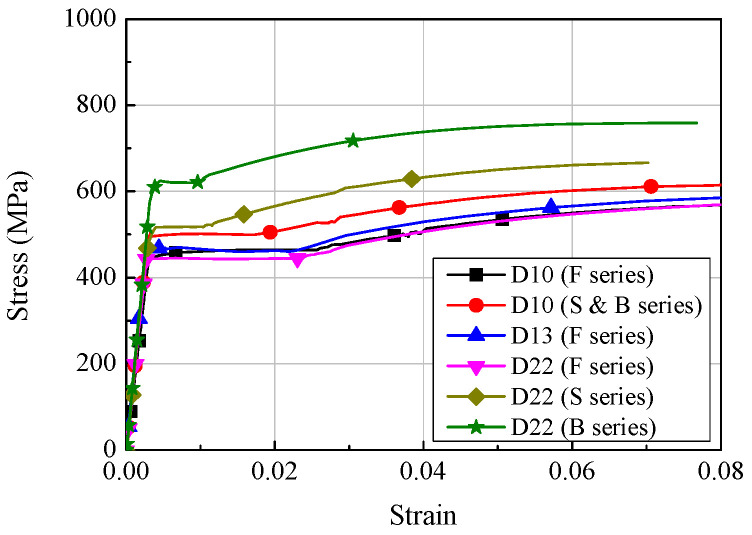
Stress-strain relationships of concrete.

Figure 2 shows the stress-strain relationships of the reinforcements used in the preparation of the specimens. For the F series, D22 and D13 were used as tensile reinforcement and compressive reinforcement, respectively, and their yield strengths were 448.7 MPa and 471.0 MPa, respectively. D10, having a yield strength of 496.6 MPa, was employed as a shear reinforcement. For the S series, D22, having a yield strength of 544.0 MPa, was used as a tensile reinforcement, and D10, which was also used for the F series, was employed as a compressive reinforcement. For the B series, D22, having a yield strength of 626.8 MPa, was used as a longitudinal reinforcement, and D10, having a yield strength of 517.3 MPa, was used as shear reinforcement. The elastic modulus of the reinforcements used to prepare the specimen was from 154 to 185 GPa.

### 2.2. Specimens

To evaluate the structural performance of the precast concrete beam members depending on the replacement ratio of ground granulated blast furnace slag, specimens of the F, S and B series, 5 for each series, were fabricated, as shown in Table 2. The F series specimens were prepared for the evaluation of the flexural performance, and the S and B specimens for the evaluation for shear and bond performance, respectively. In addition, the number in the name of the specimen means the replacement ratio of ground granulated blast furnace slag and fly ash as shown in Section 2.1.

**Figure 3:**
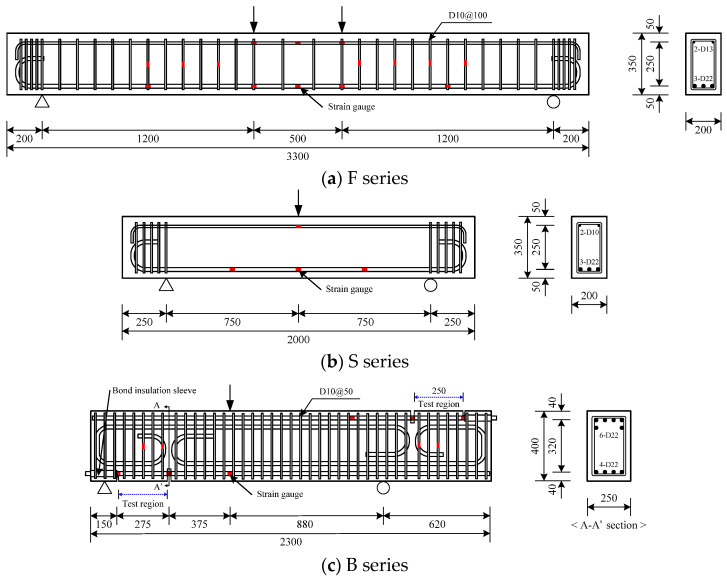
Details of specimen (unit: mm).

As shown in Table 2 and Figure 3, the F series specimens were 3300 mm long and had a cross-sectional area of 200 × 350 mm. To make the flexural failure dominant, the shear span-to-depth ratio (*a/d*) was designed to be 4.0, and thus the clear span was 2900 mm long. The F series had a tensile reinforcement ratio of 1.94%, and shear reinforcements were arranged in the experimental interval in a gap of 100 mm to prevent shear failure. The S series specimens were 2000 mm long and had a cross-sectional area of 200 × 350 mm. To induce shear failure, no shear reinforcements were arranged in the S series specimens. The B series specimens were 2300 mm long and had a cross-sectional area of 250 × 400 mm. To prevent flexural failure from preceding bond failure, two-fold longitudinal reinforcements were arranged. In addition, shear reinforcements were densely arranged with gaps of 50 mm to prevent shear failure. As shown in Figure 3, the experimental interval for the B series was designed to be 250 mm, which was the distance between the grooves to induce cracks. Because of the crack-inducing grooves, transfer of the tensile force within the experimental interval was possible only by bonding at the interface between reinforcements and concrete. To prevent the longitudinal reinforcement from being affected by the reaction force at the ends of the experimental interval, the bonds between the reinforcements and the concrete were insulated using steel pipes. For the B series, an experimental interval was designed at both the top and bottom ends of the specimens to evaluate the bond performance of the top and bottom longitudinal reinforcements. Strain gauges were attached to the tensile reinforcements, compressive reinforcements and shear reinforcements of all the specimens to measure the strain of the reinforcements.

### 2.3. Loading and Measurement Methods

**Figure 4:**
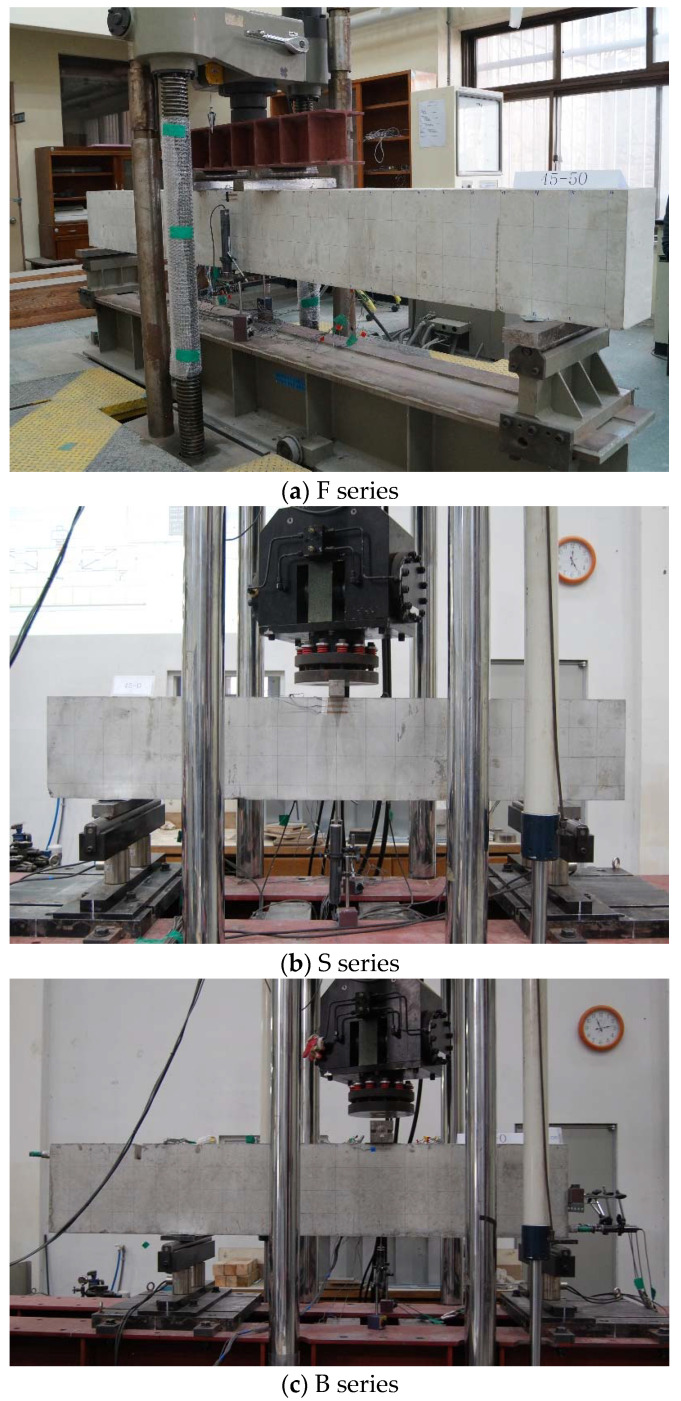
Test setup of specimen.

Loading to the specimens was performed using a universal testing machine (UTM) (Shimadzu, Kyoto, Japan) in the shape of a simple beam, as shown in Figure 4. Four-point loading was applied to the F series to induce a clear flexural interval; 3-point loading was used for the S and B series. Regardless of the specimen series, load control was applied up to the maximum load, and displacement control was applied after the maximum load until the stress decreased to 85% of the maximum load or lower.

The deflection of the beam by loading was measured by installing a linear variable differential transducer (LVDT) (Tokyo Measuring Instruments Lab., Tokyo, Japan) at the central bottom of the loading point where the maximum deformation of the specimens was generated. In addition, strain gauges for concrete were attached to positions 10 mm and 30 mm away from the compressive edge to measure the strain of the concrete at the loading point. As shown in Figure 4c, an additional LVDT was installed at the center of the longitudinal reinforcements arranged at the ends of the B series specimens to measure the slip of the inner and corner longitudinal reinforcements by bond failure.

## 3. Evaluation of Flexural Performance

### 3.1. Load-Deflection Relationships

**Figure 5:**
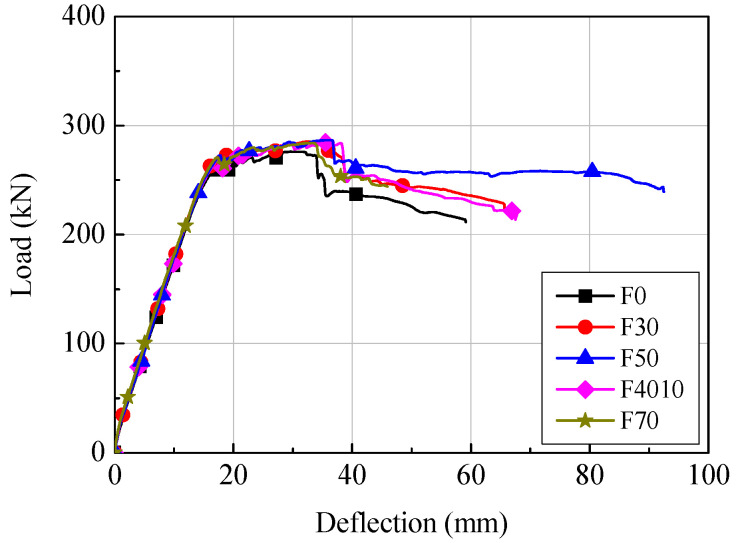
Load-deflection relationships of F series.

Figure 5 shows the load-deflection relationships of the F series specimens based on the load measured using the UTM and the displacement measured using the LVDT installed at the central bottom of the specimens. In all specimens, regardless of the replacement ratio of the ground granulated blast furnace slag, when the load was increased after the generation of flexural cracks, the tensional reinforcements reached yield strain with the growth of flexural cracks. After the maximum stress, the specimens showed typical flexural behavior in which the resisting force was decreased by the crushing of concrete at the compressive edges.

Flexural cracks of the F series specimens were generated at loads of 24.3 to 32.3 kN; the deflection was 0.46 to 1.04 mm. The tensile reinforcements reached yield strain at 216.8 to 241.5 kN, and the displacement was 12.5 to 14.1 mm. The load and deflection at the maximum stress were 276.2 to 286.7 kN and 29.7 to 36.0 mm, respectively. In the cases of the F series, the stress of all the specimens, except the F50 specimen, significantly decreased (about 10% or more) after the maximum stress. This may be because of the crushing of the concrete that happened at the compressive edges.

### 3.2. Flexural Strength

**Figure 6:**
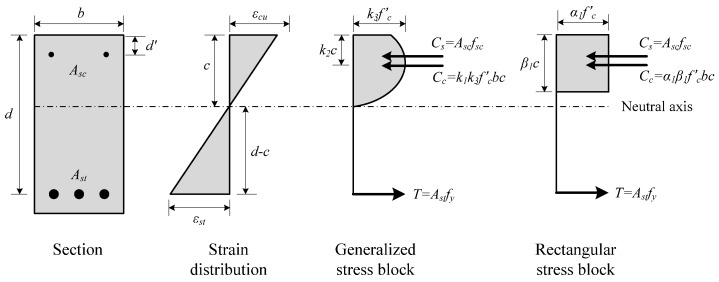
Stress distribution in the cross-section at ultimate.

Table 3 compares the flexural strength of the F series specimens between the experimental results and the analytical results. The experimental results shown in Table 3 were obtained through calculation using the load measurements from the load cell of the UTM. The analytical results were obtained using Equations (1) and (2) according to the equilibrium conditions of force and compatibility conditions of strain, as shown in Figure 6. To compare the prediction results depending on the coefficient of the equivalent stress block, the ultimate moment of the F series specimens was calculated by applying the coefficient of the equivalent stress block provided by the ACI 318-19 [25] and EC 2 [26] standards.

(1) $$M_{y}=T\left(d-\frac{c}{3}\right),$$

(2) $$M_{u}=\alpha_{1}f_{c}^{\prime }\beta_{1}cb\left(d-0.5\beta_{1}c\right)+A_{sc}f_{sc}\left(d-d^{\prime }\right),$$

Here, $M_{y}$ is the yield moment, $M_{u}$ is the ultimate moment, T is the tensile force, d is the effective death of the beam, c is the depth of the neutral axis, $\alpha_{1}$ and $\beta_{1}$ are equivalent rectangular stress block parameters (η and λ for EC 2), $f_{c}^{\prime }$ is the compressive strength of concrete, b is the beam width, $A_{sc}$ is the cross-sectional area of the compressive reinforcement, $f_{sc}$ is the stress of the compressive reinforcement and $d^{\prime }$ is the distance from the compressive edge to the midpoint of the compressive reinforcement.

As shown in Table 3, the mean ratio of the yield moment of the tensile reinforcement in the analytical results to that in the experimental results was 0.99, and the coefficient of variation was 4.32%, indicating that the experimental results were effectively predicted when the replacement ratio of the ground granulated blast furnace slag was used as a variable. With regard to the prediction of the ultimate moment based on ACI 318-19 and EC 2, regardless of the replacement ratio of ground granulated blast furnace slag, the mean ratios of the experimental results to the analytical results were 1.21 and 1.19, respectively, showing that the experimental results were predicted on the safe side. With regard to the ultimate moment, the mean ratio of the experimental results to the analytical results was lower by less than 2% and the coefficient of variation was also lower by less than 1% in the results from the EC 2 than in the results from the ACI 318-19, indicating that the prediction by the EC 2 was better.

### 3.3. Neutral Axis Depth Ratio

**Figure 7:**
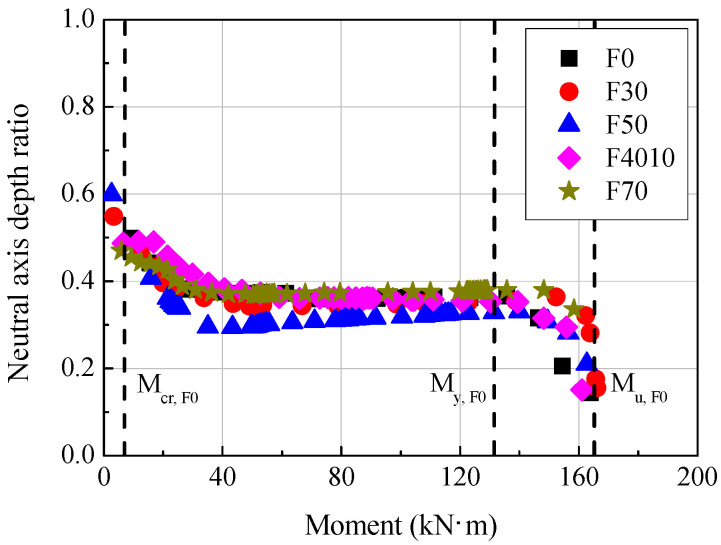
Neutral axis depth ratio-moment relationships of F series.

Figure 7 shows the change of the neutral axis depth ratio (*c/d*) depending on the replacement ratio of ground granulated blast furnace slag. The neutral axis depth in the experimental results was calculated using the strain measured by the strain gauges attached to the tensile reinforcement and the compressive edge concrete according to the Bernoulli-Euler hypothesis, as shown in Figure 6 and Equation (3):

(3) $$c=\frac{\varepsilon_{c}}{\varepsilon_{st}}\left(d-c\right)=\frac{\varepsilon_{c}}{\varepsilon_{st}+\varepsilon_{c}}d,$$

Here, $\varepsilon_{c}$ is the strain of the compressive edge concrete and $\varepsilon_{st}$ is the strain of the tensile reinforcement.

As shown in Figure 7, the neutral axis depth ratio decreased after the generation of flexural cracks, regardless of the replacement ratio of ground granulated blast furnace slag. The neutral axis depth ratio remained in a range between 0.3 and 0.4 before the yield of the tensile reinforcement, after which it began to decrease again. The neutral axis depth ratio at the ultimate was 0.15 or lower.

### 3.4. Ductility Factors

Ductility refers to the load resistance capability while maintaining the deformation of the member without a significant decrease of the stress; it is represented by a curvature ductility factor ($\mu_{\varphi }$) and a displacement ductility factor ($\mu_{\Delta }$). Table 3 compares the ductility factors of the F series specimens based on the experimental results. The curvature ductility factor and displacement ductility factor were calculated from the experimental results using Equations (4) and (5):

(4) $$\mu_{\varphi }=\frac{\varphi_{u}}{\varphi_{y}},$$

(5) $$\mu_{\Delta }=\frac{\Delta_{u}}{\Delta_{y}},$$

Here, $\varphi_{y}$ represents the yield curvature of the tensile reinforcement ($\varphi_{y}=\varepsilon_{y}/\left(d-c\right)$), $\varphi_{u}$ is the curvature when the compressive edge strain of the concrete is $\varepsilon_{cu}$ ($\varphi_{u}=\varepsilon_{cu}/c$), $\varepsilon_{y}$ is the yield strain of the tensile reinforcement, $\varepsilon_{cu}$ is the ultimate strain of the concrete, $\Delta_{y}$ is the yield displacement of the tensile reinforcement and $\Delta_{u}$ is the displacement when the compressive edge strain of the concrete is $\varepsilon_{cu}$.

As shown in Table 3, among the F series specimens, the curvature ductility factor was lowest in the F0 specimen (6.45) and highest in the F50 specimen (9.86). The F70 specimen was excluded from the analysis because the Bernoulli-Euler hypothesis was not satisfied due to an abnormality of the strain values measured in tensile reinforcement and concrete. The displacement ductility factor was also lowest in the F0 specimen (2.27) and highest in the F50 specimen (2.88). ACI 318-19 [25] secures the member ductility by applying a ductility value 2.5 times as high as the yield strain (when the yield strength of the tensile reinforcement is 400 MPa) or a higher value to the tension-controlled sections, and EC 2 [26] by limiting the value to the neutral axis depth ratio (*c/d*) (0.25 or lower for C50 at ultimate limit state). As shown in Table 3 and Figure 7, the curvature ductility factor and displacement ductility factor of the specimens prepared by substituting ground granulated blast furnace slag were generally higher than those of the reference specimen F0 by at least 28% and at least 4%, respectively. Therefore, the required ductility may be secured even if the replacement ratio of ground granulated blast furnace slag is increased to 70%.

### 3.5. Serviceability

**Figure 8:**
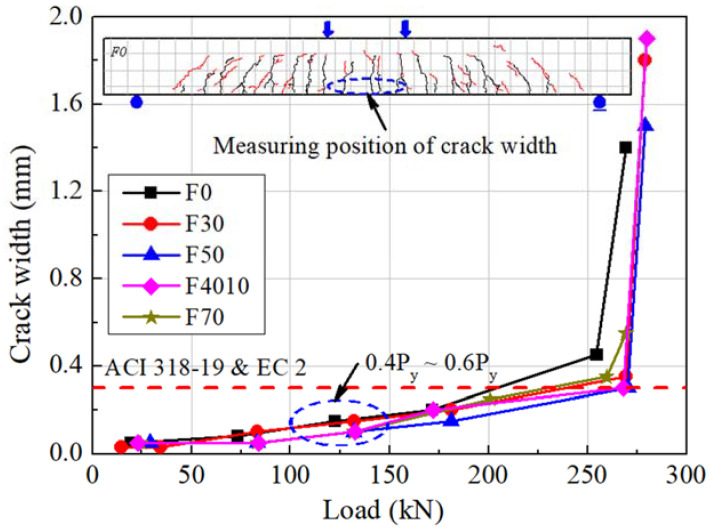
Crack width-load relationships of F series.

Figure 8 shows the course of the crack width growth in the F series specimens according to the load. The crack width was measured at the position of the tensile reinforcement between the loading points, which is the pure bending zone, and the crack that grew the largest among the crack widths measured at 3–5 places was used. The crack width was increased similarly to the increase of the load, regardless of the replacement ratio of ground granulated blast furnace slag. In the ultimate, the number of the flexural cracks observed in the experimental zone was similar, in the range between 22 and 24. The crack width in the 0.4*P_y_*–0.6*P_y_* zone, corresponding to the service load state, was less than 0.3 mm, which satisfied the allowable crack width constraints provided by ACI 318-19 [25] and EC 2 [26]. The growth of the crack width by load and the number of generated cracks were similar between the specimens fabricated by substituting ground granulated blast furnace slag and the reference specimen F0, indicating that the replacement ratio of ground granulated blast furnace slag has a negligible impact on the serviceability of the flexural members.

## 4. Evaluation of Shear Performance

### 4.1. Shear Force-Deflection Relationships

**Figure 9:**
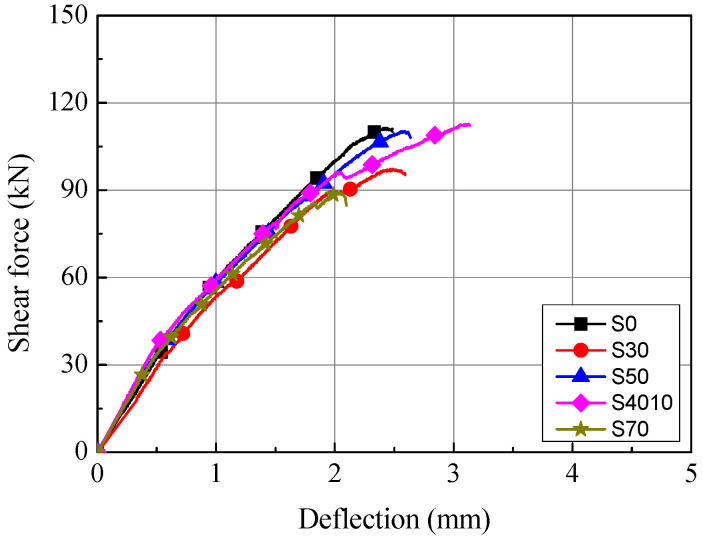
Shear force-deflection relationships of S series.

**Figure 10:**
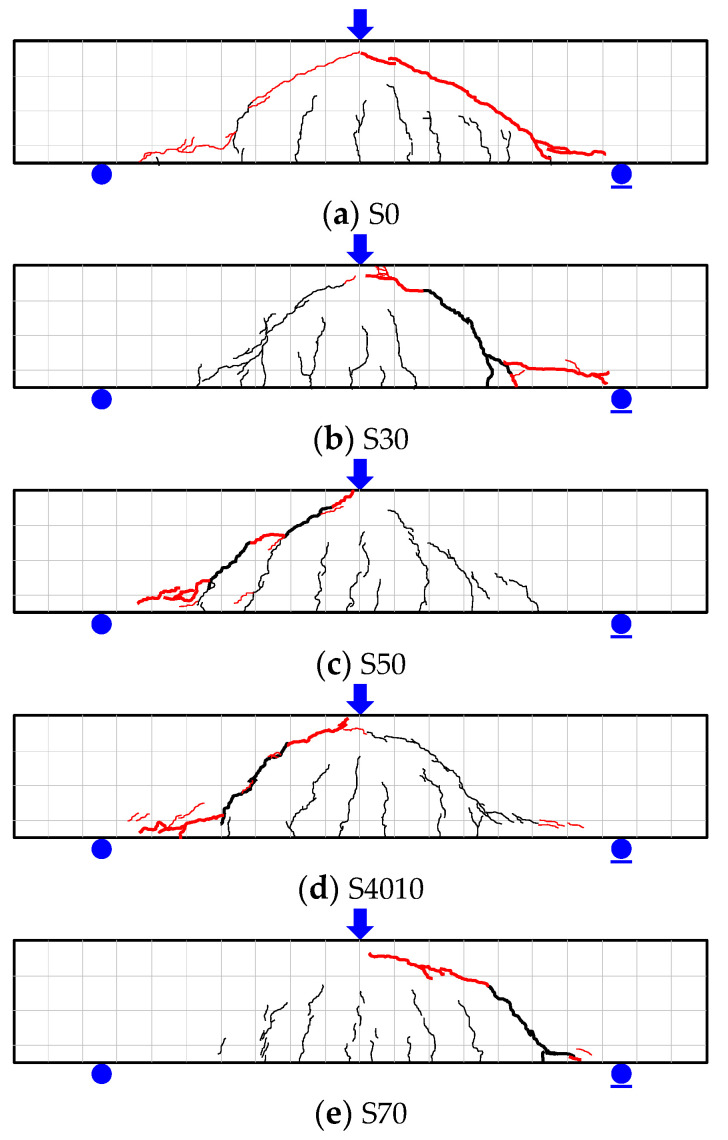
Crack patterns at failure of S series.

Figure 9 shows the shear force-deflection relationships of the S series specimens based on the load measured in the experiment and the deflection measured by the LVDT installed at the central bottom of the specimens. As shown in Figure 9 and Figure 10, flexural cracks were generated in the S series at the initial stage of loading, regardless of the replacement ratio of ground granulated blast furnace slag. As the shear force increased, diagonal cracks connecting loading and reaction positions were observed. After the generation of diagonal cracks, the S series specimens tended to resist the shear force through stress redistribution by dowel action of the tensile reinforcement, and showed shear behavior in which the stress decreased with the generation of additional diagonal cracks.

As shown in Table 4, the initial flexural cracks and diagonal cracks of the S series specimens were generated at the shear forces of 26.7 to 34.5 kN and 38.2 to 52.6 kN, respectively, for which the deflection was 0.39 to 0.57 mm and 0.60 to 0.86 mm, respectively. Therefore, the shear force and behavior were similar among the S series specimens regardless of the replacement ratio of ground granulated blast furnace slag. The maximum shear force of the S series specimens was highest in the S4010 specimen (112.8 kN) and lowest in the S70 specimen (88.8 kN). The stress of the S70 specimen was about 20% different from that of the reference S0 specimen, for which the concrete compressive strength at the maximum shear force was similar to the S70 specimen. Therefore, as the replacement ratio of ground granulated blast furnace slag increased, consideration is needed to secure the shear performance according to the decrement of the contribution by the concrete to the shear strength.

### 4.2. Comparison of Shear Strength

Table 4 compares the shear strength between the experimental results and the analytical results. The analytical results for the prediction of the shear strength, shown in Table 4, were obtained using the ACI 318-19 [25] and EC 2 [26] standards, which propose the contribution by the concrete to the shear strength, as described in Equations (6) and (7):

(6) $$V_{c,\;ACI}=\left[0.66\lambda_{s}\lambda {\left(\rho_{w}\right)}^{1/3}\sqrt{f_{c}^{\prime }}+N_{u}/6A_{g}\right]bd,$$

(7) $$V_{c,\;EC2}=\left[\left(0.18/\gamma_{c}\right)k{\left(100\rho_{w}f_{c}^{\prime }\right)}^{\frac{1}{3}}+0.15N_{u}/A_{g}\right]bd,$$

Here, $\lambda_{s}$ represents the size effect modification factor, wherein $\lambda_{s}=\sqrt{2/\left(1+0.004d\right)}\leq 1$, λ is the coefficient of lightweight concrete, $\rho_{w}$ is the ratio of tensional reinforcement, $N_{u}$ is the axial load, $A_{g}$ is the gross area of concrete section, $\gamma_{c}$ is the partial factor for concrete and $k=1+\sqrt{200/d}\leq 2$.

As shown in Table 4, the shear strength of the S series specimens was compared between the analytical and experimental results. According to ACI 318-19, the mean ratio of the experimental results to the analytical results was 1.43, and the coefficient of variation was 10.03%, indicating that the prediction was relatively conservative. According to EC 2, the mean ratio of the experimental results to the analytical results was 1.71, which was about 20% higher than that of ACI 318-19, indicating that the prediction of the experimental results was more conservative. However, EC 2 showed a coefficient of variation of 9.73%, which was about 3% lower in comparison with ACI 318-19, indicating that the accuracy of EC 2 was relatively high. In addition, comparison of the shear strength of the S series specimens between the analytical results and the experimental results confirmed that the specimens satisfied the performance required by the ACI 318-19 and EC 2 standards, regardless of the replacement ratio of ground granulated blast furnace slag. However, considering that the compressive strength of the concrete of the specimens fabricated by substituting ground granulated blast furnace slag tended to decrease in comparison with reference specimen S0 as the replacement ratio increased, it may be reasonable to set the replacement ratio of ground granulated blast furnace slag at 50% or lower to secure the shear performance.

## 5. Evaluation of Bond Performance

### 5.1. Bond Stress-Slip Relationships

**Figure 11:**
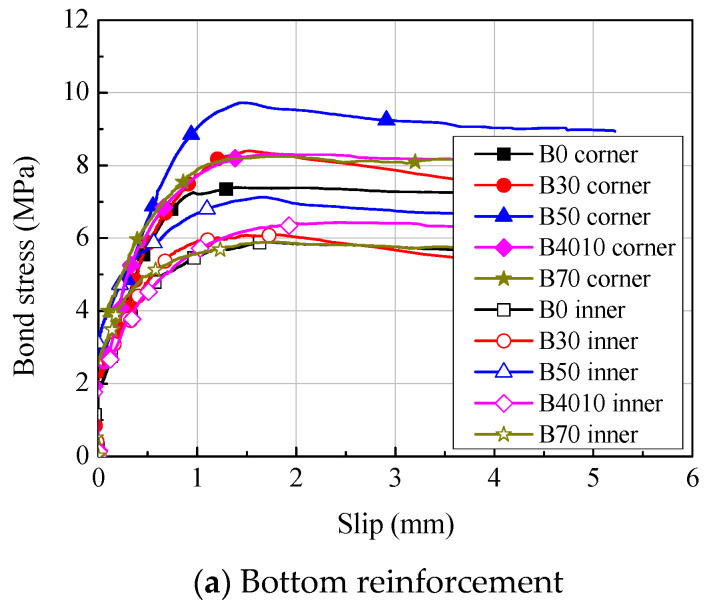

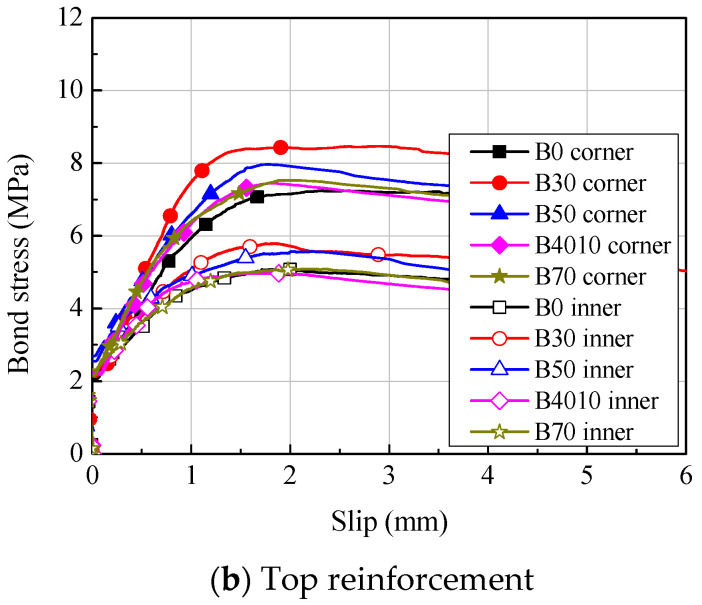
Average bond stress-slip relationships.

**Figure 12:**
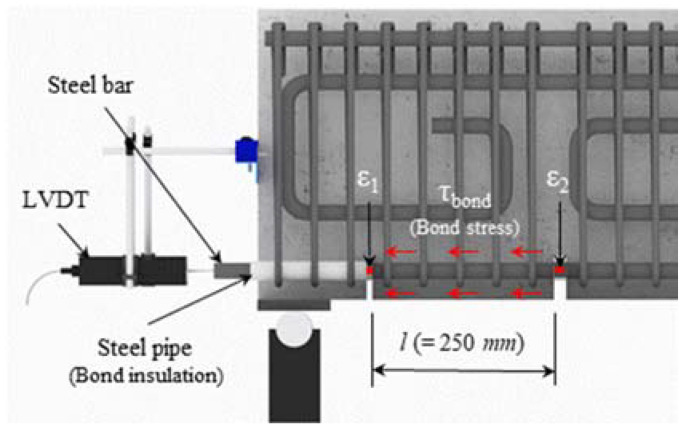
Measuring method of slip.

The bond stress-slip relationships and the bond strength of the B series specimens are shown in Figure 11 and Table 5, respectively. The bond stress of the longitudinal reinforcements was calculated using Equation (8) based on the strain distribution measured from the strain gauges attached to the crack-inducing grooves in the experimental zone, as shown in Figure 12. The slip was calculated using Equation (9), considering the elongation of the reinforcement from the value measured from the LVDTs installed at the ends of the specimens.

(8) $$\tau_{bond}=\frac{\left(\varepsilon_{2}-\varepsilon_{1}\right)E_{s}A_{st}}{\Sigma \varphi l},$$

(9) $$Slip=s_{LVDT}-\frac{\left(\varepsilon_{2}-\varepsilon_{1}\right)}{2}l,$$

Here, $\varepsilon_{1}$ is the strain of longitudinal reinforcement at the tension end, $\varepsilon_{2}$ is the strain of longitudinal reinforcement at 250 mm apart from the tension end, $E_{s}$ is the elastic modulus of the reinforcement, $A_{st}$ is the cross-sectional area of the reinforcement, Σφ is the sum of the nominal perimeter of the reinforcement, l is the the bond length and $s_{LVDT}$ is the quantity of slip in the reinforcement measured by LVDT at the end of the specimen.

**Figure 13:**
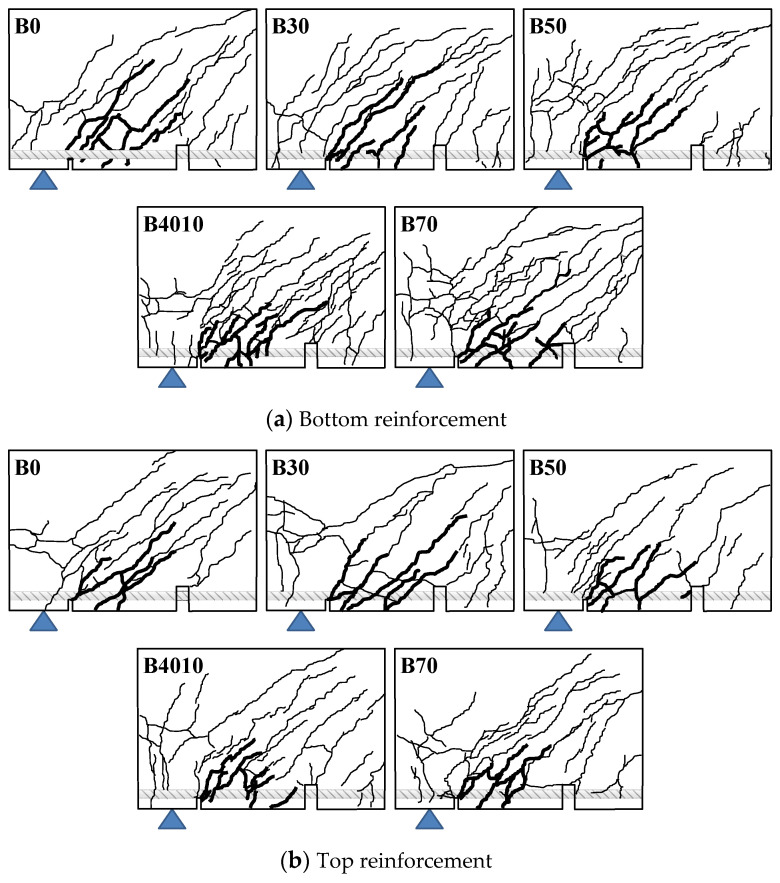
Crack patterns at failure of B series.

As shown in Figure 11 and Figure 13, regardless of the replacement ratio of ground granulated blast furnace slag, flexural cracks were found in the B series specimens at the crack-inducing grooves located at the boundary of the experimental zone in the early stage of the loading. As the load increased, flexural cracks increased, and many bond cracks were found in the experimental zone. When the maximum bond stress was reached, slip of the inner and corner longitudinal reinforcements was about 2 mm. Then, as the slip significantly increased, bond degradation also considerably occurred, decreasing the stress by bond failure.

As shown in Table 5, regardless of the replacement ratio of ground granulated blast furnace slag, the bond strength of the B series specimens was about 6.89 to 9.73 MPa at the corner longitudinal reinforcements and 4.97 to 7.13 MPa at the inner longitudinal reinforcements, the bond strength being about 37% higher at the corner longitudinal reinforcements than at the inner longitudinal reinforcements. This may be because the constraint of the transverse reinforcement provided more disadvantages to the inner longitudinal reinforcements than to the corner longitudinal reinforcements, and the overlapping of the ring-tension areas in the inner longitudinal reinforcements may have made the generation of bond cracks easier [4].

Comparison of the bond strength between the bottom reinforcements and the top reinforcements of the B series specimens showed that the mean bond strength of the bottom reinforcements was higher in all the B series specimens than those of the top reinforcements, at ratios of about 1.03 to 1.25, except for the B30 specimen. The consolidation effect of compacting during concrete placement may have increased the bond strength at the bottom reinforcements. In addition, the bond strengths in consideration of the compressive strength of the concrete ($\tau_{mean}/\sqrt{f_{c}^{\prime }}$) were compared. The ratio of the mean values of the specimens fabricated by substituting ground granulated blast furnace slag to the value of B0 were 0.84 to 1.19 and 0.90 to 1.04 at the bottom and top reinforcements, respectively. The ratio of the mean values at both the bottom and top reinforcements was 1.00, indicating that the bond performance was similar. Therefore, it was experimentally confirmed that there was no problem in force transmission between the reinforcement and the concrete, even when the replacement rate of ground granulated blast furnace slag was applied up to 70%.

### 5.2. Comparison of Bond Strength

The bond strength obtained from the experiment with the B series specimens was evaluated in comparison with the prediction results from ACI 318-19 [25], Fujii-Morita [27] and Maeda [28], as shown in Table 5. The prediction formulas according to ACI 318-19, Fujii-Morita and Maeda take into consideration the impacts of the concrete and the shear reinforcements in calculating the bond strength, as shown in Table 6.

Table 5 shows the ratio of the experimental results to the prediction results obtained from the B series specimens. Regardless of the replacement ratio of ground granulated blast furnace slag, the formula proposed by Maeda best predicted the experimental results, with a mean experimental/analytical ratio of 1.26 and a coefficient of variation of 8.8%. The mean experimental/analytical ratios according to the ACI 318-19 and Fujii-Morita formula were 1.57 and 1.47, respectively, and their coefficient of variation were 13.0% and 10.8%, indicating that these formulas underestimated the experimental results in comparison with Maeda’s formula. The coefficient of variation was about 23–48% higher, showing a relatively large deviation.

The bond strength was compared between the inner longitudinal reinforcements and the corner longitudinal reinforcements. The mean experimental/analytical ratios of the ACI 318-19, Fujii-Morita and Maeda formulas were 1.06 to 1.32 and their coefficient of variation were 7.3 to 9.6% for the inner longitudinal reinforcements; these values were 1.46 to 1.81 and 10.9 to 15.7% for the corner longitudinal reinforcements. This showed that predictions were better for inner longitudinal reinforcements than for corner longitudinal reinforcements. The difference may be because the prediction formulas did not consider the constraint by the transverse reinforcement. In addition, according to the positions of the longitudinal reinforcements, ACI 318-19 [25] evaluates the bond strength of the bottom reinforcements to be higher than that of the top reinforcements by about 30% and by about 22% in the case of Fujii-Morita [27]. Maeda [28] reflects the effect of the compressive strength of concrete and may appear differently depending on the compressive strength. However, when 50 MPa is applied, the bottom reinforcements are evaluated higher than the top reinforcements ones by about 14%. The experimental results showed that the mean bond strength of the B series specimens was about 11% higher at the bottom reinforcements than at the top reinforcements, which was similar to the prediction results. However, the absolute values of the bond strength, obtained experimentally, were lower by about 19% or more than the prediction results. The ratios of the bond strength of the bottom reinforcements to that of the top reinforcements were 1.08, 1.25 and 1.07 in the B0, B50 and B70 specimens, respectively. This suggests that the difference of the absolute bond strength between the experimental results and the prediction results may be due to an experimental error rather than being the effect of the replacement ratio of the ground granulated blast furnace slag.

## 6. Conclusions

This study was conducted to apply ground granulated blast furnace slag to precast concrete members to find an appropriate replacement ratio. The flexural, shear and bond performances of precast concrete beams were evaluated using the replacement ratio of ground granulated blast furnace slag as a variable. Although it may vary depending on the chemical composition of ground granulated blast furnace slag, the following conclusions could be obtained in this study.

- The flexural strength of the F series specimens was predicted by ACI 318-19 and EC 2. The mean ratio of the yield moment of the experimental results to that of the prediction results was 0.99, and the mean ratios of the ultimate moment were 1.21 and 1.19, respectively. This showed that the experimental results were predicted conservatively;
- The mean shear strengths of the S series specimens, measured experimentally, were 1.43 times and 1.71 times higher than the shear strength required by the ACI 318-19 and EC 2 standards, regardless of the replacement ratio of ground granulated blast furnace slag. However, in consideration of the concrete compressive strength, the shear strength of the S series specimens decreased as the replacement ratio of ground granulated blast furnace slag increased. Therefore, consideration is needed to determine the appropriate replacement ratio that can secure the required shear performance;
- The bond strengths of the B series specimens, measured experimentally, were 1.26 to 1.57 times higher than the values predicted by the formulas of ACI 318-19, Fujii-Morita and Maeda, regardless of the replacement ratio of ground granulated blast furnace slag. Therefore, the required bond strength was sufficiently satisfied. The formula proposed by Maeda predicted the experimental results at a mean experimental/analytical ratio of 1.26 with a coefficient of variation of 8.8%, indicating that the prediction was better than those obtained using formulas according to ACI 318-19 and Fujii-Morita;
- In consideration of the experimental results, in using the ground granulated blast furnace slag as an admixture for precast concrete, the cement replacement ratio may be increased up to 70% without causing any problems in securing the structural performance. However, in order to ensure sufficient safety of precast concrete members, it is considered reasonable to apply the replacement ratio of ground granulated blast furnace slag to 50% or less.

## Figures and Tables

**Table 1:** Concrete mix proportions.

Specimens	*f_ck_* (MPa)	W/B (%)	S/a (%)	Unit Weight (kg/m^3^)							
				W	C	GGBS	FA	S	G	AE	AD
C0	45	36.0	47.9	180	500	-	-	812	883	1.25	3.00
C30		34.4		172	350	150	-	817	888	1.75	
C50		32.4		162	250	250	-	827	899	2.00	
C4010		32.4		162	250	200	50	821	892	2.50	
C70		30.4		152	150	350	-	836	909	2.50	

W/B: water binder ratio, S/a: fine aggregate modulus, W: water, C: cement, GGBS: ground granulated blast furnace slag, FA: fly ash, S: fine aggregate, G: coarse aggregate, AE: air-entraining agent, AD: water reducing admixture.

**Table 2:** Properties of specimens.

Specimens		*f’_c_* (MPa)	Size (mm)			*a/d*	Reinforcement (MPa)		
			*h*	*b*	*l*		Tension	Compression	Shear
F series	F0	46.7	200	350	3300	4.0	3-D22 *f_y_* = 448.7	2-D13 *f_y_* = 471.0	D10@100 mm *f_wy_* = 496.6
	F30	58.0							
	F50	53.1							
	F4010	56.9							
	F70	45.2							
S series	S0	46.7	200	350	2000	2.5	3-D22 *f_y_* = 544.0	2-D10 *f_y_* = 496.6	-
	S30	58.0							
	S50	53.1							
	S4010	56.9							
	S70	45.2							
B series	B0	46.7	250	400	2300	2.0	6-D22 *f_y_* = 626.8		D10@50 mm *f_wy_* = 517.3
	B30	58.0							
	B50	53.1							
	B4010	56.9							
	B70	45.2							

*f’_c_*: compressive strength of concrete, *h*: height of section, *b*: width of section, *l*: length, *a/d*: shear span to depth ratio.

**Table 3:** Comparison between analytical and experimental results of F series.

Specimens	*f’_c_* (MPa)	Experimental Result								Experimental/Analytical		
		Moment		Curvature			Deflection			*M_y,exp_/M_y_*_, *ana*_	*M_u, exp_*/*M_u_*_, *ACI*_	*M_u, exp_*/*M_u, _*_*EC2*_
		*M_y_* (kN·m)	*M_u_* (kN·m)	*φ_y_* (mm^−1^)	*φ_u_* (mm^−1^)	*φu*/*φy*	Δ*_y_* (mm)	Δ*_u_* (mm)	Δ*_u_*/Δ*_y_*			
F0	46.7	133.4	165.7	1.34	8.46	6.45	13.1	29.7	2.27	0.97	1.18	1.17
F30	58.0	133.3	171.4	1.33	12.58	9.46	12.8	34.4	2.69	0.97	1.20	1.19
F50	53.1	130.1	172.4	1.31	12.92	9.86	12.5	36.0	2.88	0.94	1.22	1.20
F4010	56.9	139.0	170.8	1.36	11.24	8.26	13.6	35.6	2.62	1.01	1.20	1.19
F70	45.2	144.9	170.4	1.40	-	-	14.1	33.2	2.35	1.05	1.22	1.20
Mean										0.99	1.21	1.19
COV (%)										4.32	1.26	1.25

*M_y_*: yield moment, *M_u_*: ultimate moment, *φ_y_*: yield curvature, *φ_u_*: ultimate curvature, Δ*_y_*: deflection at yield moment, Δ*_u_*: deflection at ultimate moment.

**Table 4:** Comparison between analytical and experimental results of S series.

Specimens	*f’_c_* (MPa)	Experimental Result						Experimental/Analytical	
		At Flexural Crack		At Diagonal Tension Crack		At Peak		*V_u, exp_*/*V_u, ACI_*	*V_u, exp_*/*V_u, EC2_*
		*V_cr_* (kN)	Δ*_cr_* (mm)	*V_d_* (kN)	Δ*_d_* (mm)	*V_u_* (kN)	Δ*_u_* (mm)		
S0	46.7	34.5	0.51	52.6	0.86	111.2	2.40	1.61	1.89
S30	58.0	33.8	0.57	49.2	0.79	97.2	2.49	1.26	1.54
S50	53.1	28.2	0.41	49.4	0.80	110.2	2.58	1.49	1.80
S4010	56.9	35.4	0.48	49.4	0.76	112.8	3.10	1.48	1.80
S70	45.2	26.7	0.39	38.2	0.60	88.8	2.01	1.30	1.53
Mean								1.43	1.71
COV (%)								10.03	9.73

*V_cr_*: shear force at flexural crack, Δ*_cr_*: deflection at flexural crack, *V_d_*: shear force at diagonal tension crack, Δ*_d_*: deflection at diagonal tension crack, *V_u_*: shear force at peak, Δ*_u_*: deflection at peak.

**Table 5:** Comparison between analytical and experimental results of B series.

Specimens	*f’_c_* (MPa)	Experimental Result (MPa)			Experimental/Analytical								
					ACI 318-19 (Considering K_tr_)			Fujii-Morita			Maeda		
		τ_corner_	τ_inner_	τ_mean_	τ_corner_/τ_ana_	τ_inner_/τ_ana_	τ_mean_/τ_ana_	τ_corner_/τ_ana_	τ_inner_/τ_ana_	τ_mean_/τ_ana_	τ_corner_/τ_ana_	τ_inner_/τ_ana_	τ_mean_/τ_ana_
B0-B	46.7	7.40	5.90	6.65	1.58	1.26	1.42	1.54	1.22	1.38	1.33	1.06	1.20
B30-B	58.0	7.69	6.00	6.85	1.47	1.15	1.31	1.43	1.12	1.27	1.31	1.03	1.17
B50-B	53.1	9.73	7.13	8.43	1.95	1.43	1.69	1.89	1.39	1.64	1.70	1.25	1.47
B4010-B	56.9	6.89	5.82	6.36	1.33	1.12	1.23	1.30	1.09	1.19	1.18	1.00	1.09
B70-B	45.2	7.74	5.76	6.75	1.68	1.25	1.46	1.63	1.22	1.42	1.40	1.05	1.23
B0-T	46.7	7.24	5.08	6.16	2.01	1.41	1.71	1.83	1.29	1.56	1.49	1.05	1.27
B30-T	58.0	8.46	5.76	7.11	2.10	1.43	1.77	1.92	1.31	1.62	1.62	1.10	1.36
B50-T	53.1	7.97	5.57	6.77	2.07	1.45	1.76	1.89	1.32	1.61	1.57	1.10	1.34
B4010-T	56.9	7.32	4.97	6.15	1.83	1.25	1.54	1.68	1.14	1.41	1.41	0.96	1.18
B70-T	45.2	7.53	5.09	6.31	2.12	1.43	1.78	1.94	1.31	1.62	1.57	1.06	1.31
Mean					1.81	1.32	1.57	1.71	1.24	1.47	1.46	1.06	1.26
COV (%)					15.7	9.6	13.0	13.3	7.9	10.8	10.9	7.3	8.8

**Table 6:** Model for bond strength.

Criteria and Researchers	Formulas
ACI 318-19 [25]	$\tau_{ACI}\left(MPa\right)=\frac{1.1\lambda \sqrt{f_{c}^{\prime }}\left(\frac{c_{b}+K_{tr}}{d_{b}}\right)}{4\psi_{t}\psi_{e}\psi_{s}}$ $K_{tr}=\frac{40A_{tr}}{sN}$
Fujii-Morita [27]	$\tau_{FM}\left(MPa\right)=\tau_{co}+\tau_{st}$ $\tau_{co}=\left(0.096b_{i}+0.134\right)\sqrt{f_{c}^{\prime }}$ $\tau_{st}=7.8\frac{kA_{tr}}{sNd_{b}}\sqrt{f_{c}^{\prime }}\leq 0.27\sqrt{f_{c}^{\prime }}$ $b_{i}=\min\left(b_{vi}=\sqrt{3}\left(\frac{2C_{min}}{d_{b}}+1\right),\;b_{ci}=\sqrt{2}\left(\frac{C_{s}C_{b}}{d_{b}}+1\right)-1,\;b_{si}=\frac{b}{Nd_{b}}-1\right)$
Maeda [28]	$\tau_{MAE}\left(MPa\right)=\tau_{co}+\tau_{st}$ $\tau_{co}=\left(0.117b_{i}+0.163\right)\sqrt{f_{c}^{\prime }}$ $\tau_{st}=\left(0.110+0.096\frac{n}{N}\right)\frac{b\rho_{w}}{Nj_{t}}10^{4}\leq \left(0.365+0.322\frac{n}{N}\right)\frac{b\rho_{w}f_{wy}}{Nd_{b}}$ $b_{i}=\frac{b}{Nd_{b}}-1$

## Data Availability

The data presented in this study are available upon request from the corresponding author.
